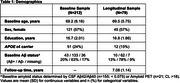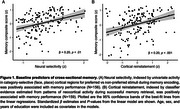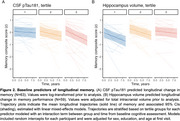# Multimodal predictors of cross‐sectional and longitudinal memory in a cognitively unimpaired aging cohort

**DOI:** 10.1002/alz70856_106059

**Published:** 2026-01-08

**Authors:** Alexandra N. Trelle, Jintao Sheng, Tammy T. Tran, Ted N. Wilson, Isha Sai, America Romero, Jennifer Park, Lucah Medina Guerra, Gayle K. Deutsch, Anthony D. Wagner, Elizabeth C. Mormino

**Affiliations:** ^1^ Stanford University School of Medicine, Stanford, CA, USA; ^2^ Stanford University, Stanford, CA, USA; ^3^ Wu Tsai Neuroscience Institute, Stanford, CA, USA; ^4^ University of Oregon, Eugene, OR, USA

## Abstract

**Background:**

Episodic memory function in aging varies considerably both across individuals and within individuals over time. Identifying factors that explain variance in memory is important for improving prediction of risk for cognitive decline in older adults. Here we examine associations of structural, functional, and molecular factors with cross‐sectional and longitudinal memory function in a normal aging cohort.

**Method:**

Participants were enrolled in the Stanford Aging and Memory Study (SAMS; *N* = 212; mean age: 69.5 ± 5.8 years, 57% female) and cognitively unimpaired at baseline. As part of an ongoing longitudinal extension of SAMS, a subset of participants (*N* = 79) have returned for longitudinal cognitive assessment (mean follow‐up: 7.08 ± 1.14 years). Memory was assessed using a composite score comprised of delayed recall subtests from Logical Memory, Hopkins Verbal Learning Test and the Brief Visual Memory Test. Baseline predictors of interest included Lumipulse CSF pTau181, hippocampus volume from manually segmented T2‐structural MRI, and fMRI measures of neural selectivity and cortical reinstatement measured during associative memory encoding and retrieval, respectively. Linear models and linear mixed effects models examined cross‐sectional and longitudinal associations, respectively, between predictors of interest and memory performance. All models included age, sex, and education as covariates.

**Result:**

All baseline predictors exhibited significant associations with age (*p* < .005). Among baseline predictors, associations were observed between reinstatement and neural selectivity (β = 0.37, *p* < .001) and between reinstatement and CSF pTau181 (β = ‐0.20, *p* < .05). Cross‐sectional effects with the memory composite were observed across both functional measures (neural selectivity: β = 0.20, *p* < .01; reinstatement: β = 0.20, *p* < .001). Associations with CSF pTau (*p* < .001) and hippocampus volume (*p* < .01) were observed when examining longitudinal change in memory over time. A trend level association between neural selectivity and longitudinal memory performance was also present (*p* = .07).

**Conclusion:**

Functional, structural, and molecular markers relevant for aging and Alzheimer's disease independently impact memory performance in human aging. Combining these measures may improve the prediction of clinically meaningful decline and elucidate factors that promote cognitive maintenance in older age.